# Infrared Radiation of Graphene Electrothermal Film Triggered Alpha and Theta Brainwaves

**DOI:** 10.1002/smsc.202200064

**Published:** 2022-11-09

**Authors:** Yanghua Lu, Renyu Yang, Yue Dai, Deyi Yuan, Xutao Yu, Chang Liu, Lixuan Feng, Runjiang Shen, Can Wang, Shenyi Dai, Qi Ge, Shisheng Lin

**Affiliations:** ^1^ College of Information Science and Electronic Engineering Zhejiang University Hangzhou 310027 P. R. China; ^2^ Hangzhou Gelanfeng Technology Co. Ltd. Hangzhou 310051 P. R. China; ^3^ Hangzhou Liangchun Technology Co. Ltd. Hangzhou 311500 P. R. China; ^4^ Hangzhou Neuro Technology Co. Ltd. Hangzhou 310051 P. R. China; ^5^ Chongqing 2D Materials Institute Chongqing 400015 P. R. China; ^6^ State Key Laboratory of Modern Optical Instrumentation Zhejiang University Hangzhou 310027 P. R. China

**Keywords:** brainwaves, electroencephalogram, electrothermal film, graphene, infrared radiation

## Abstract

The alpha and theta frequency brainwave activity in electroencephalogram (EEG) signal is proven to correlate with attention, inhibitory processes, memory, perceptual abilities, and sleep. The decreasing of brainwaves is demonstrated to be the reason of aging and even Alzheimer's disease, so triggering alpha and theta brainwave activity may bring positive behavioral modifications such as promoting health care and a quick sleep. Herein, it is discovered that infrared radiation from multilayer graphene electrothermal film can obviously promote the appearance of alpha and theta brainwaves in the human brain. In particular, the occurrence frequency and duration time of the alpha and theta waves in EEG can be effectively enhanced up to 2.3/2.9 and 3.0/4.1 times, respectively. The comparative effect of different working temperatures and heating materials is systematically investigated, indicating efficient infrared radiation from the multilayer graphene electrothermal film, which coincides with the human‐body thermal‐radiation wavelength range from 7 to 14 μm, may be the main mechanism for this enhancement. The multilayer graphene film electrical heater represents a convenient and surprising way for triggering alpha and theta brainwaves, which has many potential applications in the area of enlarged healthcare requirements.

## Graphical abstract

A multilayer graphene electrothermal film is proposed to trigger enhanced alpha and theta brainwaves in the human brain, attributing to the efficient infrared radiation from graphene located in the range from 7 to 14 μm. This work represents a convenient and noninvasive characterization tool for enhancing the activity of brain waves, which has many potential applications in the area of enlarged healthcare requirements.

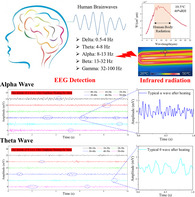

## 1 Introduction

As a 2D material, graphene has significant advantages with unique electronic, optical, thermal, and mechanical properties,^[^ 1, 2, 3 ^]^ such as excellent carrier mobility, strong electron–electron interaction, and ultrahigh thermal conductivity.^[^ 4, 5, 6 ^]^ Recently, thermal infrared emission in carbon materials, especially the graphene, has been explored,^[^ 7, 8 ^]^ which is attributed to the strong in‐plane vibrational transitions of carbon atoms.^[^ 9, 10 ^]^ While applying a bias voltage through the graphene film, most of the electronic energy can be transformed into infrared radiation, apart from Joule heat dissipated into the substrate contacted.^[^ 11, 12 ^]^ Infrared radiation is an invisible electromagnetic wave with a longer wavelength than visible light, which can be further subdivided into three different wavelengths: near‐infrared (0.8–1.5 μm), mid‐infrared (1.5–5.6 μm), and far‐infrared (5.6–1000 μm) radiation.^[^ ^13^ ^]^ While passing a current through the graphene, mid‐infrared and far‐infrared rays (4–20 μm) are radiated into free space.^[^ ^14^ ^]^ Recently, we have demonstrated the fabrication of large‐scale multilayer graphene and patented it as the multilayer graphene film with a high far‐infrared emissivity over 90%,^[^ ^15^ ^]^ which can penetrate 2–3 mm into human skin.^[^ ^16^ ^]^ Especially, the infrared radiation of graphene is matching with the human radiation wavelength (7–14 μm),^[^ ^17^ ^]^ which can substantially exert strong rotational and vibrational effects with humans at the molecular level.^[^ ^18^ ^]^ So the graphene infrared technique can be used in medical treatment and daily life, which can improve human health or heat preservation.^[^ 19, 20 ^]^

Recently, LED lights flashing at a specific 40 Hz frequency were found to significantly reduce amyloid beta plaques in the visual cortex of Alzheimer's disease mice,^[^ ^21^ ^]^ inducing gamma oscillations that help the brain in suppressing amyloid beta production and activating cells that destroy plaques.^[^ 22, 23 ^]^ These studies could prove that the a‐beta protein is not primary cause of Alzheimer's, but the weakening of brainwaves.^[^ ^21^ ^]^ The decreasing of brainwaves has been demonstrated to be the reason of aging and even Alzheimer's disease.^[^ 24, 25 ^]^ So regulating and activating the brainwaves should be an effective and potential way to improve the activity and recovery of the brain. Especially, the alpha and theta frequency brainwave activity has been correlated with attention, inhibitory processes, memory, perceptual abilities, and sleep.^[^ 26, 27 ^]^ The enhanced alpha and theta brainwave activity may bring positive behavioral modifications such as promoting healthcare and a quick sleep.^[^ ^28^ ^]^ Considering the positive effect of graphene on health and the effect of light on Alzheimer's disease,^[^ ^29^ ^]^ we tend to ask the question: what is the relationship between the effect of graphene infrared radiation on the human body and the activity of brainwaves?^[^ 30, 31 ^]^ The activity of brainwaves can be recorded with an electroencephalogram (EEG), which is generated by the oscillations of the brain electric potential that provides information about underlying neural activities in the human brain.^[^ 32, 33 ^]^ Considering the convenience, noninvasiveness, and high temporal resolution, detecting and analyzing the activity of brainwaves through a fast changing dynamic EEG is the most popular electrophysiology technique to figure out the brain's operational function and human emotion,^[^ ^34^ ^]^ which can be implemented in, for example, brain injury inspection, relief of depression, creativity, or cognitive functions research.^[^ 35, 36, 37 ^]^

Herein, dynamic EEGs with and without the heating of graphene electrothermal film have been measured, and the occurrence frequency and duration time of the alpha and theta waves in EEG can be effectively enhanced under the effect of infrared radiation from graphene electrothermal film.^[^ ^38^ ^]^ Under the bias voltage of 5.0 V, the graphene film can be heated with a controllable temperature from 40 to 60 °C assisted by the feedback of negative temperature coefficient (NTC) thermistor in the graphene film. Obvious infrared radiations with wavelengths ranging from 4 to 20 μm from the multilayer graphene film with an area of 16 cm × 5 cm can be detected, which has a central peak spectrum located between 7 and 14 μm with radiation energy accounting for more than 50%. As the infrared radiation spectrum of graphene film covering the human‐body thermal‐radiation wavelength range from 7 to 14 μm,^[^ ^17^ ^]^ these infrared radiations from the graphene film can be effectively absorbed by the human skin, which can substantially exert strong rotational and vibrational effects at the molecular level. With tightly pressing a graphene electrothermal film electrical heater embedded in a scarf on the back of neck, the infrared radiations from the graphene film can effectively irradiate into the human skin.^[^ 39, 40 ^]^ The dependence of the enhancement effect on the working temperature of the graphene electrothermal film is systematically investigated. In particular, the occurrence frequency of the alpha and theta waves in the EEG can be effectively enhanced up to 2.3 and 3.0 times under the optimized temperature of 50 °C, and the duration time of the alpha and theta waves in EEG can also be extended for 2.9 and 4.1 times. The heating effects of water, Cu, and monolayer graphene are also compared with multilayer graphene film, indicating the infrared radiation from the graphene electrothermal film has the greatest enhancement effect of alpha and theta brainwaves. Although the detailed mechanism needs to be further explored, it may be related with the efficient infrared radiation from graphene locating at a range from 7 to 14 μm, coinciding with the human‐body thermal‐radiation wavelength. The controllable effect of the graphene electrothermal film on the appearance of alpha and theta waves represents a convenient and noninvasive characterization tool for the regulation and activation of human brainwaves, which has potential applications such as sleep problem treatment.

## 2 Results and Discussion

**Figure 1:**
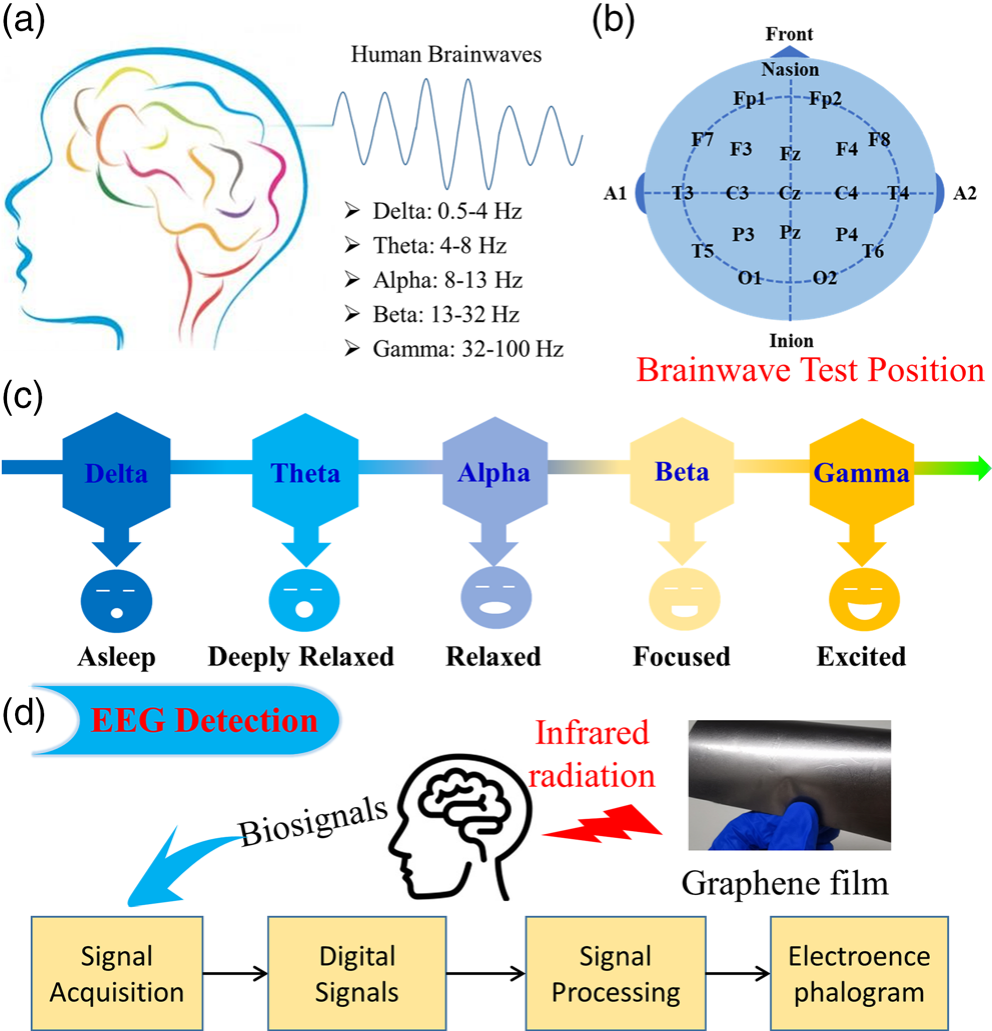
a) Schematic diagrams of EEG detection of human brainwaves. b) Human brainwaves and the frequency of five type brainwaves. c) Brainwaves test position. d) Different emotion states of five type brainwaves. General view of EEG's signal processing mode and flow diagram of signal detection.

The study of the EEG is of particular interest to discover the relationship between behaviors and brain activity. In fact, the source of the EEG is the multitude of neurons and connections between them, and more precisely, it is situated on the most external layers of the brain, known as cerebral cortex. The frequencies of brain waves detected in an EEG range from 0.5 to 100 Hz, and their characteristics are highly dependent on the degree of activity of the brain's cerebral cortex. Nowadays, EEG has become a powerful tool for clinical diagnosis of acute brain disorders, and EEG signals are subdivided into five different parts: delta (0.5–4 Hz), theta (4–8 Hz), alpha (8–13 Hz), beta (13–32 Hz), and gamma (32–100 Hz) brainwaves, as shown in **Figure** **1**a. An EEG is obtained using electrodes placed on the human scalp. There are 19 general positions on the human head for a brainwave test: Fp1, Fp2, F3, F4, F7, F8, Fz, T3, T4, T5, T6, C3, C4, Cz, P3, P4, Pz, O1, and O2, as shown in Figure 1b. These five brainwaves can be collected in the different positions, which represent “asleep”, “deeply relaxed”, “relaxed”, “focused”, and “excited”, respectively, as shown in Figure 1c. The general view of the EEG's signal processing mode and flow diagram is shown in Figure 1d. The human brain signals collected with electrodes are transformed to digital signals, and then filtered with balance electrodes signals to eliminate noise signals, outputting effective EEG signals under the effect of the graphene film.

**Figure 2:**
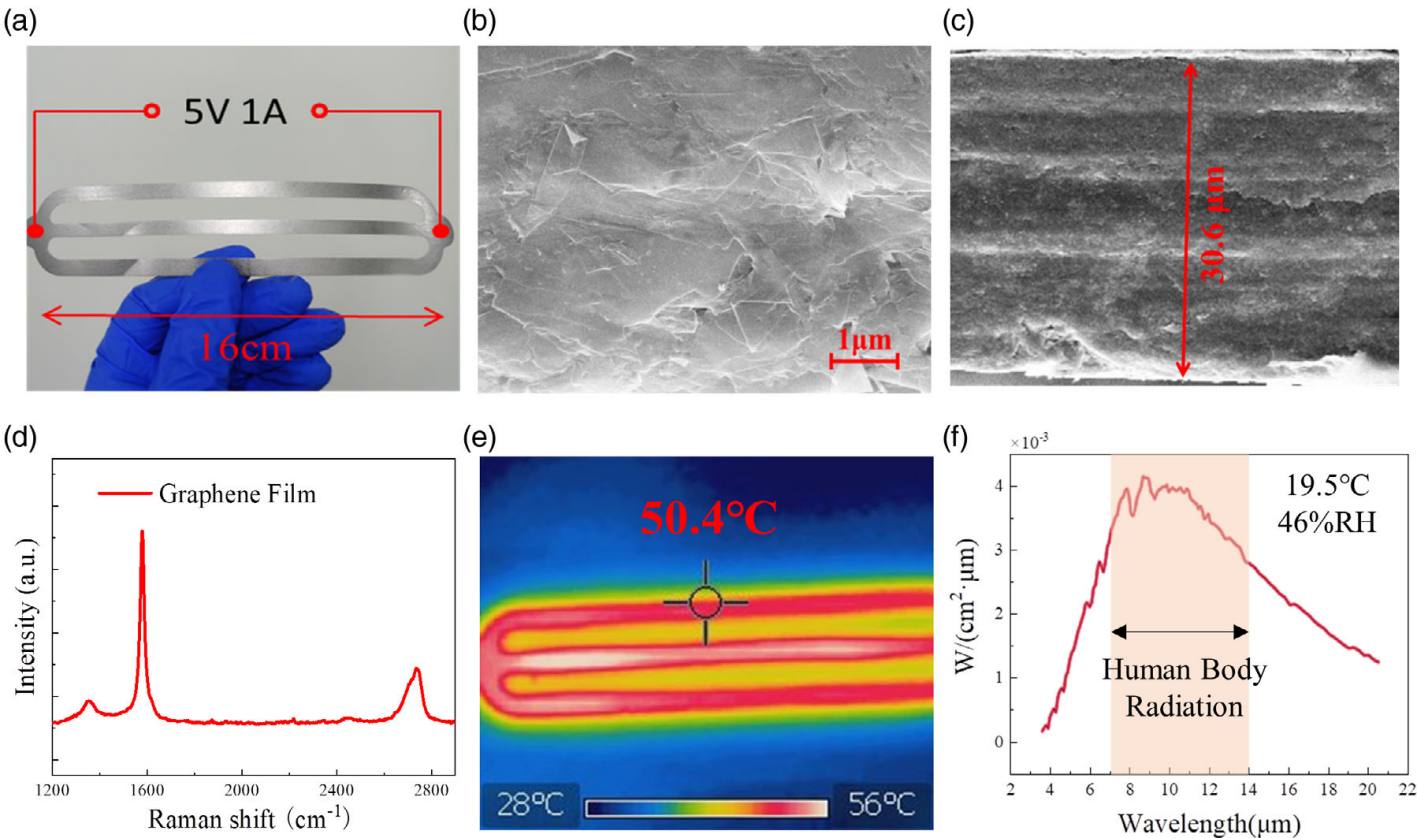
a) Optical and electrical properties of the graphene electrothermal film. b,c) Optical picture of the grid‐shaped graphene electrothermal film. d) SEM images of the graphene film in the horizontal section in (b) and the vertical section in (c). e) Raman spectrum of the graphene film at a room temperature of 25 °C. f) Infrared image of the graphene film under a bias voltage of 5.0 V. Infrared radiation spectrum of the graphene electrothermal film.

During the measurement of EEG signals, a graphene electrothermal film electrical heater was tightly pressed to the back of the neck, which was embedded in a scarf. As shown in **Figure** **2**a, a grid‐shaped graphene electrothermal film with the area of 16 cm × 5 cm was used. The graphene film fabricated by the tape casting method has excellent flexibility and mechanical stability, which indicates its superiority for a wearable heating device.^[^ 41, 42 ^]^ The top and cross‐sectional views of the scanning electron microscopy (SEM) image of the graphene film are shown in Figure 2b,c, respectively, which reveal that the graphene film was flat and composed of vertically stack graphene flake layers less than ten layers. The Raman spectrum of the graphene film at 25 °C was also measured and shown in Figure 2d, which has three peaks named as D‐peak (1348 cm^−1^), G‐peak (1584 cm^−1^), and 2D‐peak (2724 cm^−1^). In particular, the weak D‐peak showed the high quality and limited crystal defects of the graphene film. Under a bias voltage of 5 V, a temperature of 40 to 60 °C can be achieved under the feedback of NTC thermistor in the graphene film, as shown in Figure 2e. Broad infrared radiation can be detected at wavelengths between 4 and 20 μm,^[^ 14, 15 ^]^ which can penetrate 2–3 mm into human skin through the scalp,^[^ ^16^ ^]^ as shown in Figure 2f. Especially, the peak energy of the infrared radiation spectrum of graphene film covers the human‐body thermal‐radiation wavelength range from 7 to 14 μm, with radiation energy accounted for more than 50% as shown in the shaded area of Figure 2f, so the infrared radiation from the graphene film can be effectively absorbed by the human skin. As shown in Figure S1 and S2, Supporting Information, a graphene electrothermal film of 50.7 °C embedded in a scarf with a temperature of 41.8 °C was tightly pressed to the back of the neck, and the temperature of the human skin was lifted from 37.5 to 40.2 °C, attributed to the infrared radiations from the graphene film.

**Figure 3:**
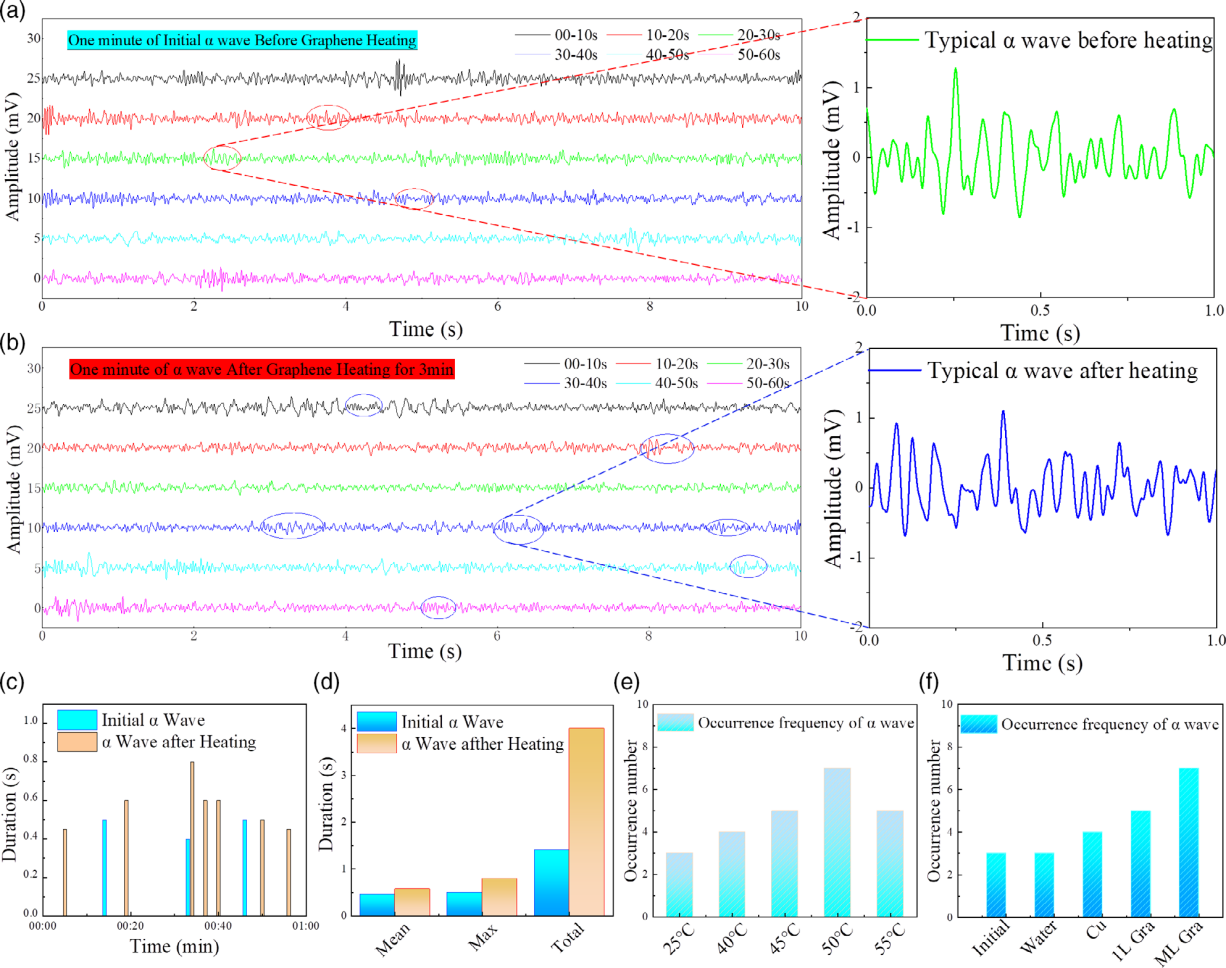
a) Signal analysis of alpha waves in EEG signals. b) One minute of initial alpha waves from O1 or O2 positions before graphene heating. Inset: Typical alpha wave before graphene heating. c) One minute of alpha waves from O1 or O2 positions after the heating effect of graphene electrothermal film for 3 min Inset: Typical alpha wave after heating. d) Detailed occurrence time of alpha wave in EEG signals for 1 min before and after heating of graphene electrothermal film. e) Occurrence time contrast of alpha wave in EEG signals before and after heating of graphene electrothermal film. f) Occurrence frequency of alpha wave in EEG signals under different heating temperature. Occurrence frequency of alpha wave in EEG signals under the heating of water, Cu, monolayer graphene, and multilayer graphene electrothermal film.

Alpha brainwaves are considered to be the brainwave state of relax and calmness, which can be measured with an EEG.^[^ ^43^ ^]^ For the alpha wave test, point O1 and point O2 were chosen to record the electric signal, and the GND and REF worked as a contrast zero potential or reference potential. The “O” of O1 and O2 means occipital lobe, an area of the human brain associated with vision. According to the brainwaves test position in Figure 1b, we found these two points place on the occipital bone at the back of the brain. The measurements were carried out in a perfectly quiet and dark room. During the measurement, the participants always closed their eyes, which avoided interruption of the visual system and reduced the noise information generated by visual processing in the occipital lobe. With tightly pressing a graphene electrothermal film electrical heater imbedded in the scarf on the back of neck, the infrared radiation from the graphene film can effectively irradiate into the human skin. The alpha wave of EEG with duration of 60 s was measured before and after the heating of graphene electrothermal film for 3 min, as shown in **Figure** **3**a,b, respectively. We discovered that the occurrence frequency of the alpha waves in EEG effectively increased from 3.0 to 7.0 times min^−1^ after participants are exposed to the infrared radiation from graphene electrothermal film. The typical alpha wave before and after graphene heating is shown in the inset of Figure 3a,b, respectively. We mainly referred to two items in data analysis: the occurrence frequency and the active time of the alpha waves before and after the heating of graphene electrothermal film. The detailed occurrence time of alpha waves in EEG signals for 1 min before and after heating of the graphene electrothermal film is shown in Figure 3c. It can be found that the mean time, maximum time, and total time of alpha waves in EEG signals were effectively increased from 0.5/0.5/1.4 to 0.6/0.8/4.0 s, as shown in Figure 3d. The rationality of the existence of alpha brainwaves is one of the basic states of the brain, which is innate in our human brain but easily hampered by the stresses of environment and life, leading to stress and anxiety diseases. The alpha waves in EEG signals indicate less anxiety and stress, leading to higher immunity and creativity.

Furthermore, the dependence of the enhancement effect on the working temperature of the graphene electrothermal film was systematically investigated. As shown in Figure 3e, the occurrence frequency of alpha waves under the temperature of 25/40/45/50/55 °C was 3.0/4.0/5.0/7.0/5.0 times min^−1^, respectively. The occurrence frequency increased and then decreased with the increase of temperature, and 50 °C was found as the optimal choice, in which the human brain can achieve the most comfortable state. To eliminate the environmental influence, we have carried out experiments on the relationship between the occurrence frequency and different heating material, such as water, Cu film, and even monolayer graphene film. As shown in Figure 3f, the occurrence frequency of alpha brainwaves under the heating of water, Cu, monolayer graphene, and multilayer graphene at 50 °C was 3.0, 4.0, 5.0 and 7.0 times min^−1^, respectively. To control the variables in the test, the resistance of the Cu, monolayer graphene, and multilayer graphene used was 5.0 Ω, as shown in Figure S3, Supporting Information. The detailed signal analysis of alpha waves in EEG signals under the heating of water, Cu, and monolayer graphene electrothermal film is shown in Figure S4, Supporting Information. Compared with the multilayer graphene electrothermal film used, the water, Cu, and monolayer graphene electrothermal film demostrated limited enhancement effect, indicating the most obvious role of infrared radiation from the multilayer graphene electrothermal film in the enhancement effect of alpha brainwaves. The infrared radiation of monolayer graphene is much weaker than the multilayer graphene, and peak energy of the infrared radiation spectrum of copper is located at wavelengths between 16 and 20 μm, which is much different from the human body.^[^ 44, 45, 46 ^]^ The typical alpha waves in EEG signals before and after the heating of Cu, monolayer graphene, or multilayer graphene electrothermal film are shown in Figure S5, Supporting Information. The power spectral density of the alpha waves in the EEG signals before and after heating of the graphene electrothermal film were also compared. As shown in Figure S6, Supporting Information, with the heating of the graphene electrothermal film, the power spectral density of alpha wave was enhanced and the peak frequency shifts to lower part. Especially, the occurrence of alpha waves in the frequency range of 8–11 Hz indicates the human brain was blankly confused and consciousness, which was gradually moving toward deeply relaxed.^[^ 35, 36 ^]^ So the heating of graphene electrothermal film can help to calm down and promote a deeper sense of relaxation and contentment.^[^ 47, 48 ^]^

**Figure 4:**
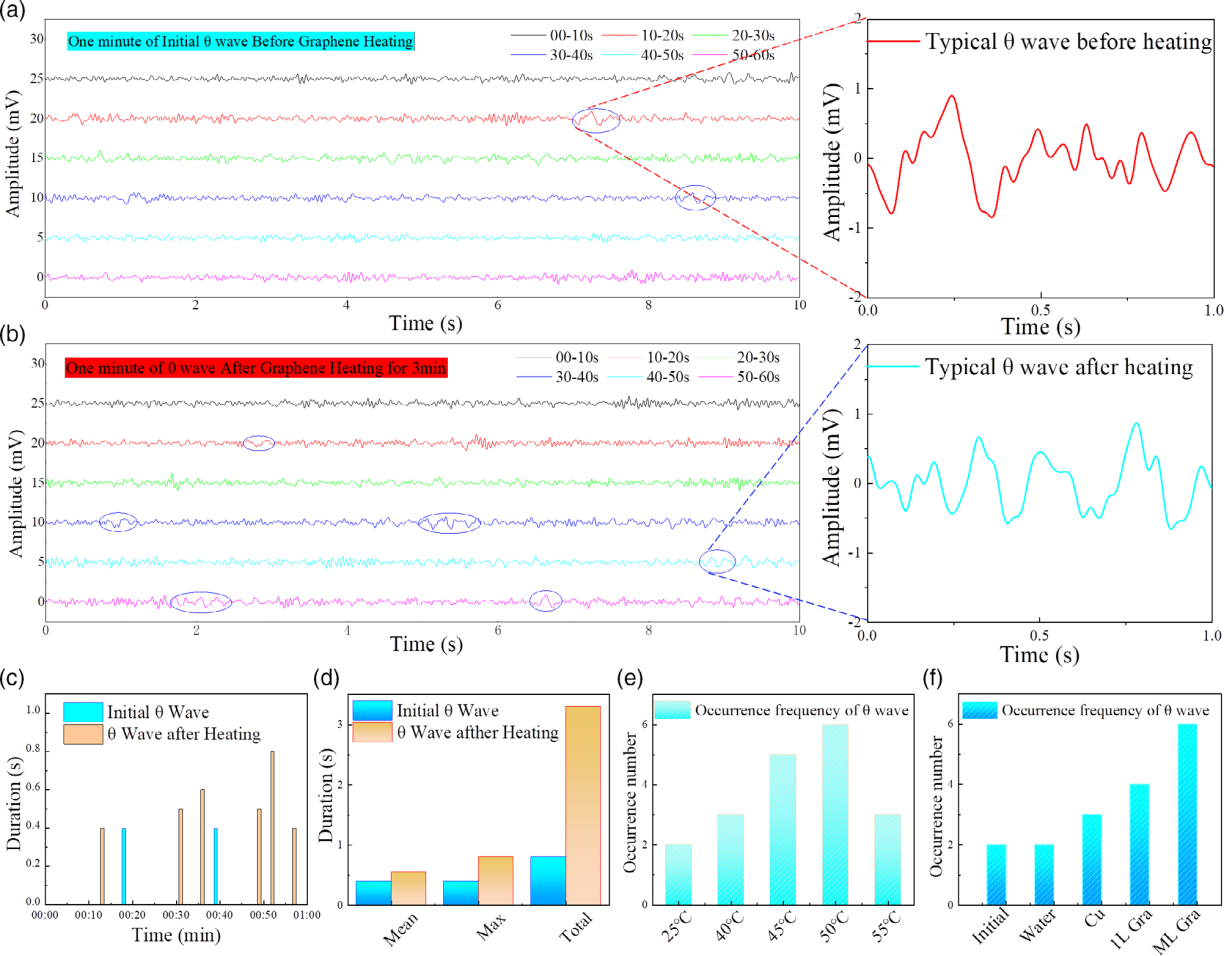
a) Signal analysis of theta wave in EEG signals. b) One minute of initial theta wave from F3 or F4 positions before graphene heating. Inset: Typical theta wave before graphene heating. c) One minute of theta wave from F3 or F4 positions after the heating of graphene film for 3 min Inset: Typical theta wave after graphene heating. d) Detailed occurrence time of theta wave in EEG signals for 1 min, before and after heating of graphene electrothermal film. e) Occurrence time contrast of theta wave in EEG signals before and after heating of graphene electrothermal film. f) Occurrence frequency of theta wave in EEG signals under different heating temperature. Occurrence frequency of theta wave in EEG signals under the heating of water, Cu, monolayer graphene, and multilayer graphene electrothermal film.

Alpha waves are the “frequency bridge” between conscious mind (beta waves) and unconscious mind (theta waves). Theta waves usually occur when deeply relaxed or near the time fall sleepin such as in trance or hypnosis, which are also important for triggering deep memories and strengthening long‐term memories. So triggering theta waves in the brain makes it easier for patients to receive hypnosis, which can be widely used in sleep or mental health related treatments. Therefore, the theta waves of EEG were also measured before and after the heating of graphene electrothermal film. As alpha and theta waves are always found in different areas, point F3 and point F4 were chosen to record the electric signal for the theta wave test, and point Cz worked as the reference potential. For the convenience of the theta wave EEG signals detection, the participants always closed the eyes and breathed regularly. To assist in the observation of the theta waves, the participants were required to hold a deep breath and keep the breath frequency in 13 to 15 times min^−1^. As theta waves are infrequent in adults, closed eyes and deep breathing can reduce the influence of the environment and enhance the occurrence of theta waves. As shown in **Figure** **4**a,b, the occurrence frequency of the theta waves in EEG was effectively enhanced from 2.0 to 6.0 times min^−1^, under the effect of infrared radiation from graphene electrothermal film. The typical theta waves before and after graphene heating are shown in the inset of Figure 4a,b, respectively. The heating of graphene electrothermal film can help to promote a deeper sense of relaxation and falling asleep, inducing more theta waves. To reveal the enhancement effect of the graphene film on the theta waves, we mainly referred to two items in data analysis: the occurrence frequency and the lasting time of the theta waves before and after the heating of graphene electrothermal film. The detailed occurrence time of theta wave in EEG signals for 1 min before and after heating of graphene electrothermal film is shown in Figure 4c. It can be found that the mean time, maximum time, and total time of theta waves in EEG signals were effectively enhanced from 0.4/0.4/0.8 to 0.6/0.8/3.3 s, as shown in Figure 4d.

Furthermore, the dependence of the theta waves enhancement effect on the working temperature of graphene electrothermal film was systematically investigated. As shown in Figure 4e, the occurrence frequency of theta waves under the temperature of 25/40/45/50/55 °C was 2.0/3.0/5.0/6.0/3.0 times min^−1^, respectively. The occurrence frequency increased and then decreased with the increase of temperature, and 50 °C was found as the optimal choice, in which the human brain achieved the most comfortable state for sleeping. For comparison, we also have carried out experiments on the relationship between the occurrence frequency and different heating material, such as a water Cu film and monolayer graphene film. As shown in Figure 4f, the occurrence frequency of theta brainwaves under the heating of water, Cu, monolayer graphene, and multilayer graphene at 50 °C was 2.0, 3.0, 4.0, and 6.0 times min^−1^, respectively. To control variables in the test, the resistance of the Cu, monolayer graphene, and multilayer graphene used was 5.0 Ω. The detailed signal analysis of theta waves in the EEG signals under the heating of water, Cu, and monolayer graphene electrothermal film is shown in Figure S7, Supporting Information. Compared with multilayer graphene electrothermal film, the water, Cu, and monolayer graphene electrothermal film demonstrated limited enhancement effect, indicating the key role of infrared radiation from the multilayer graphene electrothermal film in the enhancement effect of theta brainwaves. The typical theta waves in the EEG signals before and after the heating of Cu, monolayer graphene, or multilayer graphene electrothermal film are shown in Figure S8, Supporting Information. The power spectral density of theta waves in the EEG signals before and after heating of graphene electrothermal film was also compared. As shown in Figure S9, Supporting Information, with the heating of the graphene electrothermal film, the power spectral density of the theta waves was enhanced and the peak frequency decreased. Especially, the occurrence of theta waves during the frequency of 4‐6 Hz indicates the human mind was deeply relaxed and hypnagogic.^[^ 35, 36 ^]^ Theta waves can improve the creativity, wholeness, and intuition, which can also involve in the restorative sleep.^[^ 47, 48 ^]^ Therefore, the graphene electrothermal film electrical heater represents a convenient and noninvasive characterization tool for the regulation and activation of human brain biosignals, which has potential applications in human brain activity promotion and sleep problem treatment.

## 3 Conclusion

We have discovered that the occurrence frequency and duration time of the alpha and theta waves in the human brain can be effectively enhanced by tightly pressing a graphene electrothermal film electrical heater embedded in a scarf at the back of the neck. We have uniquely pointed out that the occurrence frequency of alpha and theta waves in the EEG can be effectively increased up to 2.3 and 3.0 times under the optimized temperature of 50 °C, and the duration time of the alpha and theta waves in EEG can also be extended effectively for 2.9 and 4.1 times. The heating effects of water, Cu, and even monolayer graphene are also compared with multilayer graphene film, indicating the key role of infrared radiation from the graphene electrothermal film in the enhancement effect of alpha and theta brainwaves. Although the detailed mechanism needs to be further explored, it may be related with the efficient infrared radiation from graphene locating at range from 7 to 14 μm, coinciding with the human‐body thermal‐radiation wavelength. The graphene electrothermal film electrical heater represents a convenient and noninvasive characterization tool for enhancing the neurocognitive function associated with systems such as memory and attention and also the detection of EEG, which has many potential applications in the area of enlarged healthcare requirements.

## 4 Experimental Section

4.1

4.1.1

##### Device Fabrication and Measurement

Before the test, the participants’ heads were treated with alcohol wipes to remove excessive sebum and keratin. All participants gave written informed consent before enrollment in this study and were screened for contraindications to EEG. Our exclusion criteria comprised the presence of a history of any neurological or psychiatric disease, use of active drugs, and any skin condition that could be worsened by the EEG detection. All experiments were conducted in accordance with ethical committee instruction and approval of the Zhejiang University. The Au disk electrodes with diameter of 10 mm were placed on the matched points. All electrodes were fixed on the scalp with medical tape and conductive paste. We got the digital signals from iNeuro system and dealt with raw data by using MATLAB (2019a, MathWorks, USA). For the alpha wave test, the participants’ foreheads were treated with alcohol wipes, the same as point O1 and point O2. The points we chose were REF, GND, O1, and O2. REF and GND were on either side of the forehead, which were acting as a contrast zero potential or reference potential. During the test, the participants should close their eyes and keep awake. Every single test lasted about 10 min, of which the last 8 min were the data of using the infrared radiation. The alpha and theta waves are always found in different areas. In the theta wave test, we chose Cz as reference point and F3 and F4 as test points. The data were dealt with in the same method as the alpha wave test, while the test process had a bit of difference. To assist the observation of the theta rhythm, the participants were required to hold deep breath in the test, and keep the breath frequency in 13–15 times min^−1^. The monolayer graphene was grown using the chemical vapor deposition (CVD) method and then transferred to a poly(ethylene terephthalate) (PET) substrate. Then, the interdigital Ag electrodes array and leads were coated on the monolayer graphene. The graphene membrane was fabricated with the tape casting method, in which the few‐layer graphene powder was dispersed in water uniformly by the ultrasonic method, before heating and drying in a PET film. The ohmic electrode of the graphene membrane was fabricated with silver epoxy and drying with hot plate at a temperature of 110 °C. Then, a plain conductor was connected to the silver electrode.

##### Characterization Analysis

The iNeuro system includes two main software, iNeuro. Client. Collect.exe (V3.1.0) and iNeuro. Client. Replay.exe (V3.0.3). The former is used for EEG collecting, and another could replay the stored EEG data. The operating system could set data collecting parameter and do some general processing, like changing the data flow, setting EEG leads and filter. We got the raw data as digital signals from iNeuro system. We selected the channels we used in test and then imported the data into MATLAB. After the signal filtering processing, effective EEG signals can be achieved. The waveforms we got were bandpass filtered at 3–20 Hz by MATLAB's “filter” function. The EEG power spectral density (PSD) is a frequency‐domain parameter, which directly reflects the values of different frequencies in the frequency domain. We used P‐welch function in MATLAB to quantify the brainwave data after preprocessing, and got the PSD graphs of alpha rhythm and theta wave from each EEG dataset. As for the parameters in P‐welch function, we set 10 s Hanning window, 5120 points of Nfft,512 Hz sampling frequency, and 50% overlap.

## Conflict of Interest

The authors declare no conflict of interest.

## Author Contributions

S.L. designed the experiments, participated the experiments, analyzed the data, conceived the study, and wrote the paper. Y.L., R.Y., and Y.D. assisted to design and carry out the experiments, and discussed the results. D.Y., X.Y., C.L., L.F., R.S., C.W., and S.D. discussed the results and assisted with experiments. All authors contributed to the preparation of the manuscript.

## Supporting information

Supplementary Material

## Data Availability

The data that support the findings of this study are available from the corresponding author upon reasonable request.
