# Supplementary material for: Infrared Radiation of Graphene Electrothermal Film Triggered Alpha and Theta Brainwaves
Source: Small Sci. 2022 Nov 9;2(12):2200064. doi: 10.1002/smsc.202200064 (PMC11935869; doi:10.1002/smsc.202200064)
Supplement: Supplementary file 1 — Supplementary Material [file SMSC-2-2200064-s001.pdf]

Supplementary Materials for

Infrared Radiation of Graphene Electrothermal Film Triggered Alpha and Theta Brainwaves

*Yanghua Lu, Renyu Yang, Yue Dai, Deyi Yuan, Xutao Yu, Chang Liu, Lixuan Feng, Runjiang Shen, Can Wang, Shenyi Dai, Qi Ge and Shisheng Lin**

Dr. Y. H. Lu, R. Y. Yang, D. Y. Yuan, X. T. Yu, C. Liu, L. X. Feng, R. J. Shen, C. Wang, Prof. S. S. Lin

College of Information Science and Electronic Engineering, Zhejiang University, Hangzhou, 310027, P. R. China.

Dr. Y. H. Lu, Y. Dai, Prof. S. S. Lin

Hangzhou Gelanfeng Technology Co. Ltd, Hangzhou, 310051, P. R. China.

Dr. Y. H. Lu, Prof. S. S. Lin

Hangzhou Liangchun Technology Co. Ltd, Hangzhou, 311500, P. R. China.

Dr. S. Y. Dai

Hangzhou Neuro Technology Co. Ltd, Hangzhou, 310051, P. R. China.

Q. Ge, Prof. S. S. Lin

Chongqing 2D Materials Institute, Chongqing, 400015, P. R. China.

Prof. S. S. Lin

State Key Laboratory of Modern Optical Instrumentation, Zhejiang University, Hangzhou, 310027, P. R. China.

Email: shishenglin@zju.edu.cn

*Corresponding author.

This file includes:

Figs. S1 to S9

Figure S1.

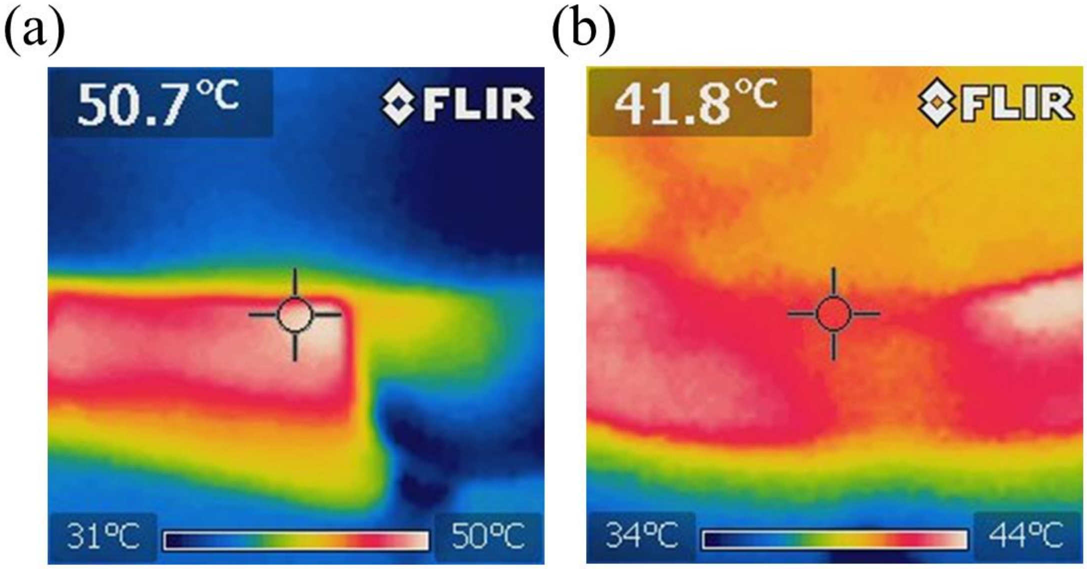

Figure S1. Infrared image of the scarf embedded with a graphene electrothermal film. (a) Infrared image of the graphene film. (b) Infrared image of the scarf.

Figure S2.

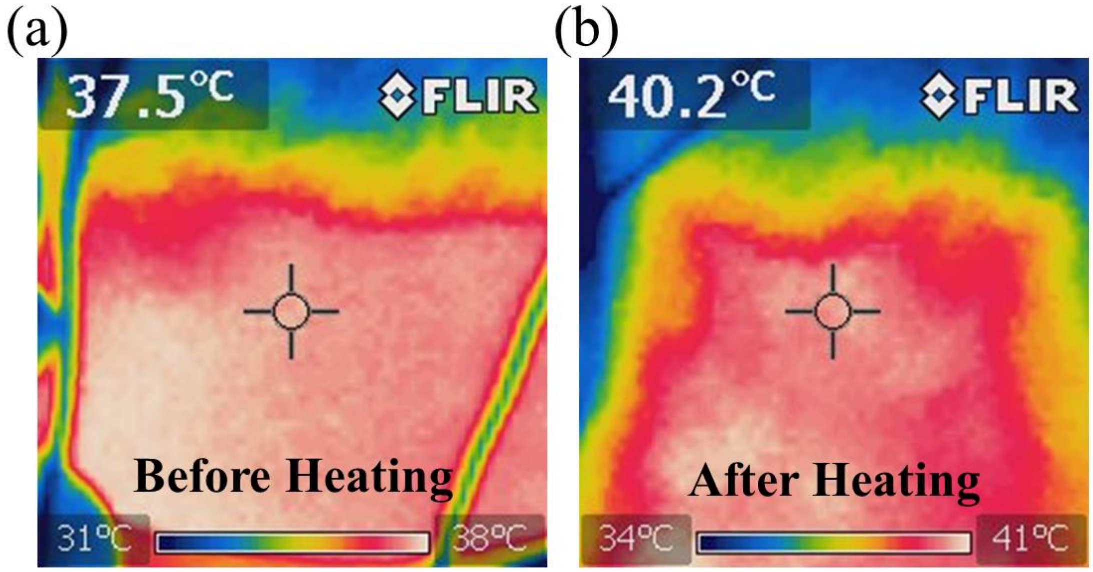

Figure S2. Infrared image of the human skin in the back of neck. (a) Infrared image of the human skin of neck before heating. (b) Infrared image of the human skin of neck after heating.

Figure S3.

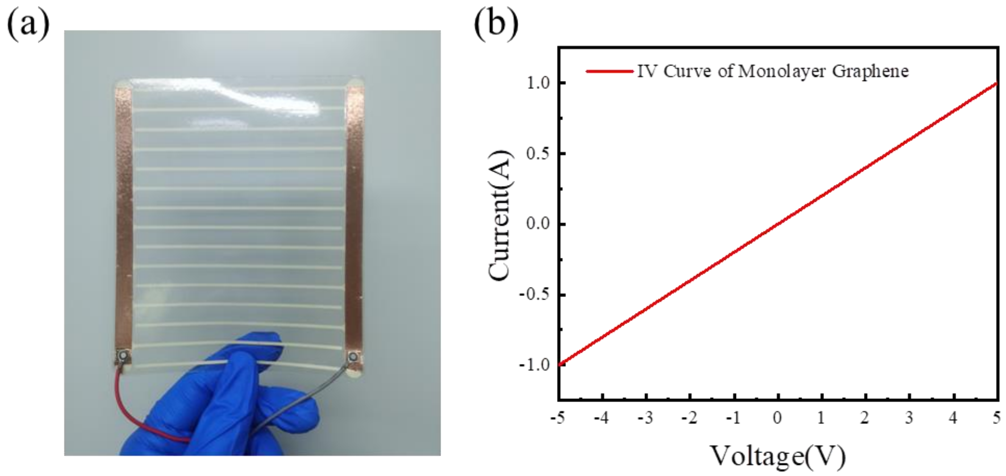

Figure S3. Optical picture and resistance of monolayer graphene electrothermal film. (a) Optical picture of the monolayer graphene electrothermal film with interdigital electrode array. (b) The IV curve of the monolayer graphene electrothermal film.

Figure S4.

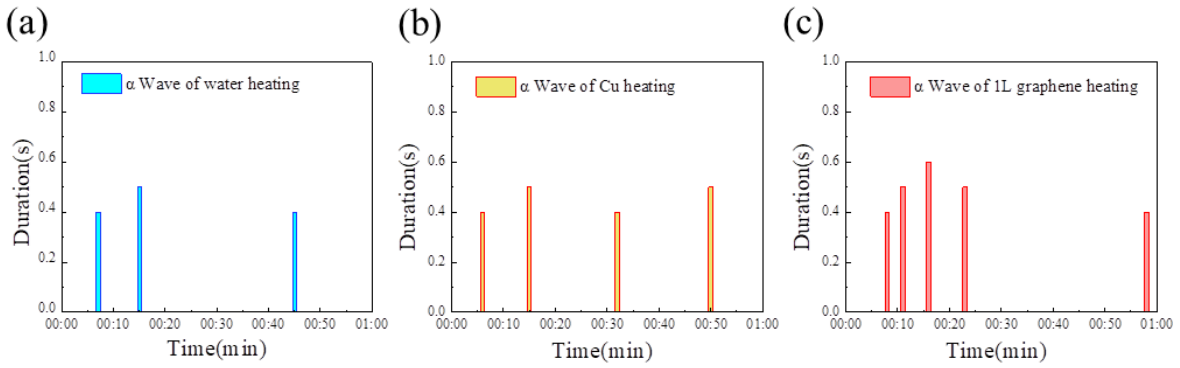

Figure S4. Signal analysis of alpha waves in EEG signals under the heating of water, Cu and monolayer graphene electrothermal film. (a) Occurrence frequency of alpha wave in EEG signals under the heating of water for one minute. (b) Occurrence frequency of alpha wave in EEG signals under the heating of Cu film for one minute. (c) Occurrence frequency of alpha wave in EEG signals under the heating of monolayer graphene film for one minute.

Figure S5.

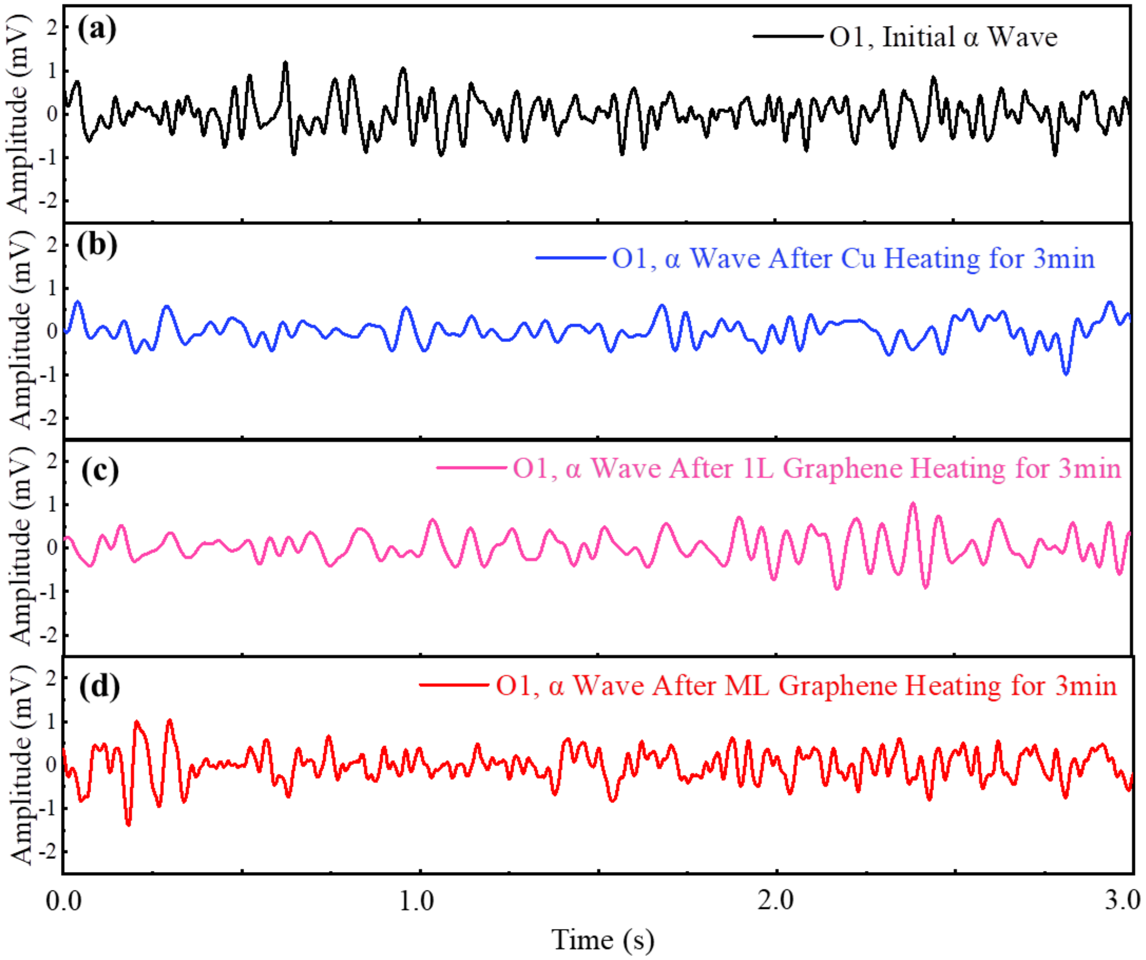

Figure S5. Typical alpha waves in EEG signals. (a) Initial alpha wave. (b) Alpha wave under the heating of Cu electrothermal film for 1min and 3min. (c) Alpha wave under the heating of monolayer graphene electrothermal film for 3min. (d) Alpha wave under the heating of multilayer graphene electrothermal film for 3min.

Figure S6.

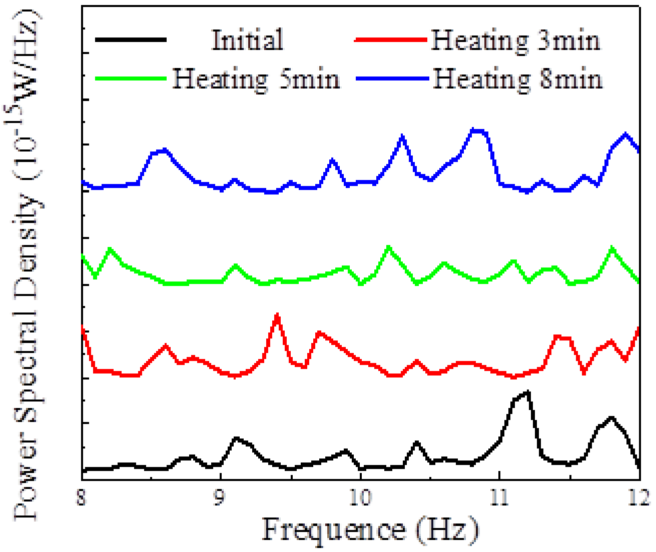

Figure S6. Power spectral density of alpha waves in EEG signals before and after heating of graphene electrothermal film in different time.

Figure S7.

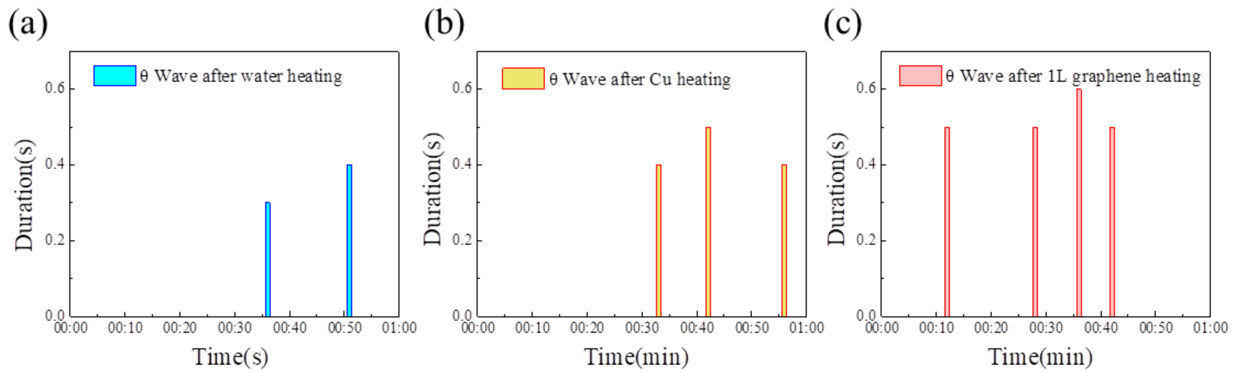

Figure S7. Signal analysis of theta waves in EEG signals under the heating of water, Cu and monolayer graphene electrothermal film. (a) Occurrence frequency of theta wave in EEG signals under the heating of water for one minute. (b) Occurrence frequency of theta wave in EEG signals under the heating of Cu film for one minute. (c) Occurrence frequency of theta wave in EEG signals under the heating of monolayer graphene film for one minute.

Figure S8.

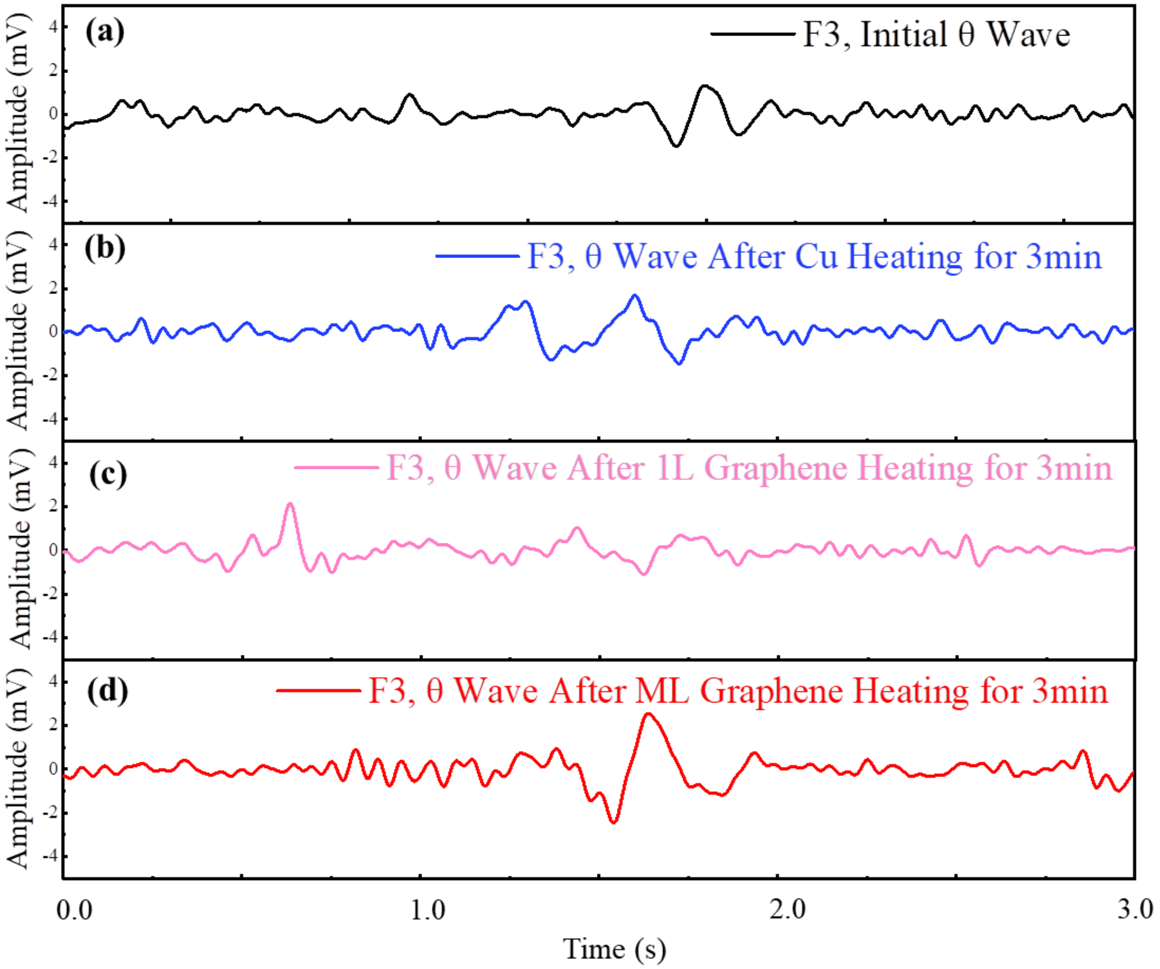

Figure S8. Typical theta waves in EEG signals. (a) Initial theta wave. (b) Theta wave under the heating of Cu electrothermal film for 1min and 3min. (c) Theta wave under the heating of monolayer graphene electrothermal film for 3min. (d) Theta wave under the heating of multilayer graphene electrothermal film for 3min.

Figure S9.

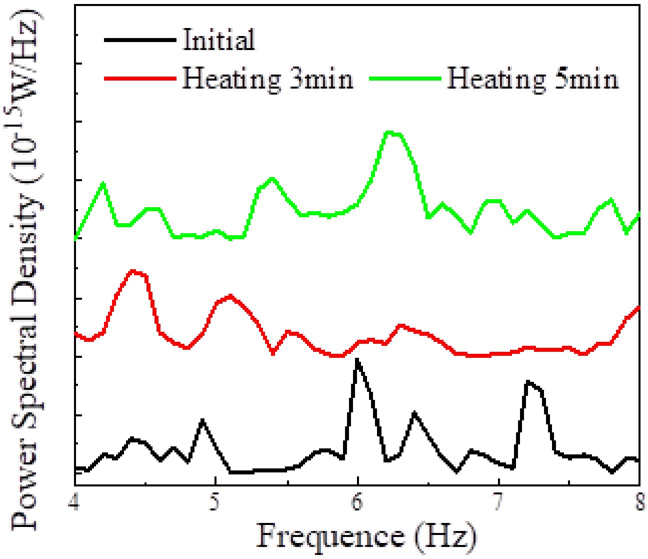

Figure S9. Power spectral density of theta waves in EEG signals before and after heating of graphene electrothermal film in different time.
